# Towards a Phenomenology of Technologically Mediated Moral Change: Or, What Could Mark Zuckerberg Learn from Caregivers in the Southern Netherlands?

**DOI:** 10.1007/s10699-015-9450-y

**Published:** 2015-11-16

**Authors:** Tamar Sharon

**Affiliations:** Philosophy Department, Maastricht University, Maastricht, Netherlands

**Keywords:** Techno-moral change, Mediation, Surveillance, Privacy, Care, Social media

## Abstract

Kamphof offers an illuminating depiction of the technological mediation of morality. Her case serves as the basis for a plea for modesty up and against the somewhat heroic conceptualizations of techno-moral change to date—less logos, less autos, more practice, more relationality. Rather than a displacement of these conceptualizations, I question whether Kamphof’s art of living offers only a different perspective: in scale (as a micro-event of techno-moral change), and in unit of analysis (as an art of living oriented to relations with others rather than the relation to the self). As a supplement and not an alternative, this modest art has nonetheless audacious implications for the ethics of surveillance.

Kamphof’s inspiring text seeks to contribute to the recent burgeoning literature in the philosophy of technology and empirical ethics that attends to the ways in which new technologies mediate not only human behavior and perception, but also morality. By highlighting the practical, mundane, and relational dimensions of the co-shaping of technology and morality, her analysis might be seen as a feminist corrective to the overemphasis on deliberation and reflection (logos) in Swierstra’s account of “techno-moral change”, and an overemphasis on the self (autos) in Verbeek’s non-humanist ethics. These rather heroic archetypes of technologically mediated moral change, Kamphof argues, are fragmentary. They miss, in Swierstra’s case, the *performance* of new fits between technology and morality, not just the deliberation over them, and in Verbeek’s case, an understanding of human being as *relational* and other-oriented, not just self-oriented. It is arguable whether either Swierstra’s or Verbeek’s theories really require this corrective, or if this is more a case of enriching our understanding of the phenomenon of technologically mediated moral change by highlighting different nodes and levels of a complex mechanism.

In Swierstra’s theory of techno-moral change morality primarily exists in the form of all but taken for granted practical routines. It is only when established norms and values become problematic, resulting for example from the introduction of a new technology, that morality moves from the realm of the self-evident to the arena of intentional reflection and argumentation—i.e., “ethics”. Ethics is thus a highly reflective affair, but it is not *solely* a reflective affair, as Kamphof at times seems to suggest. In Swierstra’s framework conscious deliberation leads to active experimentation, which can lead to more conscious deliberation and so forth, until a new fit is forged between morality and technology, during which values, if ever so slightly, are modified and can settle. The dance between these two types of grappling with the disruption of the moral landscape is the ethical storm that precedes the moral calm. It isn’t clear that this differs in any fundamental way from Schön’s “reflective conversation”, in which “professionals try out something, while listening to the talk-back of the situation”, that Kamphof suggests better describes how the caregivers in her case experimented with telemonitoring technology. Are Kamphof’s caregivers really less reflective than Swierstra’s ethics entails? They too engaged in conscious deliberation: as they “discussed working with the system” in “follow up meetings”, as they “decided” which information to communicate to clients, as they “opted” to ignore or not the data they received, and “reflected” on privacy problems. The re-articulation of privacy that they engaged in also comprised an important element of conscious deliberation. There is a danger here of misportraying Swierstra’s ethics as more deliberative than it is, and of misportraying the caregivers’ “art” as more active than it is.

However, there *is* an important difference between these two framings that Kamphof’s analysis does highlight; not of kind, but of scale. Where Swierstra’s examples, from the contraceptive pill to organ transplantation, deal with large-scale events of techno-moral change, on quite a broad socio-historical level, we might understand the case analyzed by Kamphof as a micro-event of techno-moral change, insofar as the value of privacy has effectively been modified in this case, but for a specific population, i.e., these professionals, in a specific context, i.e., caregiving for the frail elderly in the southern Netherlands. It would certainly be interesting to explore the relationship between micro- and macro-events in the aim of developing a phenomenology of technologically mediated morality.

With Verbeek, Kamphof’s disagreement hinges on the centrality given to subjectivity, to the self, within his theory. The art of living with technology is not an individual or an individualizing project, according to Kamphof, quite simply because humans are through and through relational beings, whose identity is formed in relationship to the beings around them. What this means for an art of living with technology is that it seeks to shape ethical relationships with others, more than an ethical relationship with oneself, as evidenced by the careful and sensitive attempts to re-articulate privacy in the work of the caregivers: this was about establishing good relationships with others (clients/patients), not about stylizing their own subjectivity. Implied in this corrective to Verbeek are two claims. One about the origin of ethically engaging with techno-moral change (relationality), and one about the orientation or aim of this practice (good relationships).

Concerning the first, I don’t think it is fair to use the “relationality” card against Verbeek. The philosophical frameworks he draws on, phenomenology and post-structuralism/Foucault, both imply a relational understanding of human being and an understanding of subjectivity as a by-product, an effect of relations. How, then, does the question of the aim of an art of living with technology suggested by Kamphof diverge from Verbeek’s? For Verbeek the central question of his ethics of technology is “what kind of mediated moral subjects do we aspire to be?” Stylizing *that* subject is the aim of an art of living with technology. For Kamphof, the question is rather “what kind of relationships with others (perhaps first and foremost human, rather than technological, others) do we want to forge?” These inquiries seem to be fundamentally different, but might this just be a question of which actor within the network of relations we choose to focus on, which of the relationships (to the self, to an other, to a technology), as well as which kind of technology is in question? Kamphof’s actors (and technology) of choice are not just relational because they are human beings, they are from the outset oriented towards relationality; their function is an entirely other-oriented one: care. But this does not mean that at the same time they are not asking what kind of subjects they want to be—subjects, say, who are sensitive to others’ sense of privacy, subjects who are committed to establishing good relationships. Conversely, the case of Verbeek’s technology of choice, the obstetric ultrasound, could be seen in light of the question “what kind of relationship with others do we want to forge?” Do we want the relationship between prospective parents and fetuses to be a relationship of choice? Of responsibility over life and death? Etc.

Stylization of the self and a stylization of good relationships are surely not mutually exclusive as central questions of the art of living ethically with technology. In his later works, Foucault actually offers a fruitful source for conceptualizing their intersection. While technologies of the self in classical Antiquity did have as an immediate goal the care of the self, he argues that this care of the self was also a precondition for being able to care for others.

Thus the modest art of living that Kamphof describes and seeks to conceptualize in this paper may be seen as a supplement more than a corrective to existing conceptualizations, by zooming in on a micro-event of techno-moral change and by shifting the unit of analysis or orientation of ethical engagement from the relationship to the self to the relationship with others. But it is a very promising supplement! As Kamphof suggests, the re-articulation of privacy that the caregivers undertook and the pragmatic solutions they devised are of value beyond the context of healthcare.

At a time when the creation, collection and processing of personal data is nearly a ubiquitous phenomenon and the call to “protect privacy” is the standard answer to the concerns that living in a surveillance culture raises, what added value does the shift of scale and of orientation advocated by Kamphof have? What lessons could we learn from the activity of these caregivers, and how could they be carried over into the world of social media, online shopping and the NSA? Certainly, one lesson to draw from Kamphof’s case is the idea that no judicial, objective definition of privacy (particularly one based solely in data and privacy protection laws) is exhaustive of the subjective and lived experiences of the value of privacy across various contexts. Here Kamphof’s identification of no less than six (!) meanings of privacy in practice may begin to answer what surveillance and privacy theorists dauntingly call the “privacy paradox” (Regan 2003; Nissenbaum 2009), i.e., the distance between people’s expressed concerns about privacy and their actual practices of disclosure, by showing that these are not two sides of a coin, but convey different things, under the label of privacy. Kamphof’s “zooming in” to the contextual, everyday articulations and enactments of privacy among caregivers and clients, then, takes one large step towards enriching the concept of privacy in a way that can help make the privacy paradox intelligible. Furthermore her plea to orient the art of living with technology towards the establishment of good relationships could and should, arguably, be made the focal point of surveillance ethics. The question of why surveillance is undertaken, by whom and how it may be employed, is a question of a relationship between surveiller and surveilled. Needless to say the more or less transparent, benevolent and symmetrical that relationship will be, different meanings of privacy and ways of safeguarding it in practice will emerge. While it might be idealistic or naïve to call for a transposition of the predominant value of the realm of healthcare onto the realm of corporate profit—Facebook will never really relate to me as a caregiver to a frail elderly person—what Kamphof suggests is more feasible: a transposition of pragmatic solutions that worked within one context onto the other. The next step in experimenting with a modest art of living with technology is experimenting with these solutions in other contexts.

## References

[CR1] Nissenbaum H (2009). Privacy in context: Technology, policy and the integrity of social life.

[CR2] Regan PM (2003). Privacy and commercial use of personal data: Policy developments in the United States. Journal of Contingencies and Crisis Management.

